# Enforced sialyl‐Lewis‐X (sLeX) display in E‐selectin ligands by exofucosylation is dispensable for CD19‐CAR T‐cell activity and bone marrow homing

**DOI:** 10.1002/ctm2.280

**Published:** 2021-02-23

**Authors:** Diego Sánchez‐Martínez, Francisco Gutiérrez‐Agüera, Paola Romecin, Meritxell Vinyoles, Marta Palomo, Néstor Tirado, Samanta Romina Zanetti, Manel Juan, Michela Carlet, Irmela Jeremias, Pablo Menéndez

**Affiliations:** ^1^ Department of Biomedicine, Josep Carreras Leukemia Research Institute, School of Medicine University of Barcelona Barcelona Spain; ^2^ Servei d'Immunologia Hospital Clínic de Barcelona Barcelona Spain; ^3^ Department of Apoptosis in Hematopoietic Stem Cells, Helmholtz Center Munich German Center for Environmental Health (HMGU) Munich Germany; ^4^ Department of Pediatrics Dr von Hauner Children's Hospital, LMU Munich Germany; ^5^ Department of Biomedicine, School of Medicine University of Barcelona Barcelona Spain; ^6^ Centro de Investigación Biomédica en Red‐Oncología (CIBERONC) Instituto de Salud Carlos III Madrid Spain; ^7^ Institució Catalana de Recerca i Estudis Avançats (ICREA) Barcelona Spain

**Keywords:** BM homing, CAR T‐cells, E‐selectin ligands, exofucosylation

## Abstract

CD19‐directed chimeric antigen receptors (CAR) T cells induce impressive rates of complete response in advanced B‐cell malignancies, specially in B‐cell acute lymphoblastic leukemia (B‐ALL). However, CAR T‐cell‐treated patients eventually progress due to poor CAR T‐cell persistence and/or disease relapse. The bone marrow (BM) is the primary location for acute leukemia. The rapid/efficient colonization of the BM by systemically infused CD19‐CAR T cells might enhance CAR T‐cell activity and persistence, thus, offering clinical benefits. Circulating cells traffic to BM upon binding of tetrasaccharide sialyl‐Lewis X (sLeX)‐decorated E‐selectin ligands (sialofucosylated) to the E‐selectin receptor expressed in the vascular endothelium. sLeX‐installation in E‐selectin ligands is achieved through an *ex vivo* fucosylation reaction. Here, we sought to characterize the basal and cell‐autonomous display of sLeX in CAR T‐cells activated using different cytokines, and to assess whether exofucosylation of E‐selectin ligands improves CD19‐CAR T‐cell activity and BM homing. We report that cell‐autonomous sialofucosylation (sLeX display) steadily increases in culture‐ and *in vivo*‐expanded CAR T cells, and that, the cytokines used during T‐cell activation influence both the degree of such endogenous sialofucosylation and the CD19‐CAR T‐cell efficacy and persistence *in vivo*. However, glycoengineered enforced sialofucosylation of E‐selectin ligands was dispensable for CD19‐CAR T‐cell activity and BM homing in multiple xenograft models regardless the cytokines employed for T‐cell expansion, thus, representing a dispensable strategy for CD19‐CAR T‐cell therapy.

## Graphical abstract

Glyco‐engineered enforced sialofucosylation of E‐selectin ligands was dispensable for CD19‐CAR T‐cell activity and BM homing in multiple xenograft models, thus representing an unnecessary strategy for CD19‐CAR T‐cell therapy

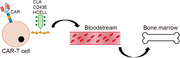

## 1 INTRODUCTION

Immunotherapy has revolutionized cancer treatment. Adoptive cell immunotherapy using T cells genetically redirected to a tumor‐specific antigen by chimeric antigen receptors (CAR) has induced impressive rates of complete response in advanced B‐cell malignancies, especially in B‐cell acute lymphoblastic leukemia (B‐ ALL).^1^, ^2^ Unfortunately, however, CAR T‐cell‐treated patients eventually progress due to either poor CAR T‐cell persistence and/or disease relapse. Common limitations associated to CD19‐CAR T‐cell treatment, but extendable to other CARs are: (i) failure to achieve complete remission, (ii) relapse with potential antigen loss, (iii) cytokine release syndrome (CRS) and related toxicities, and (iv) existence of multitreated patients not eligible for CAR T‐cell therapy due to low counts of T cells.^3^, ^4^, ^5^

In the clinical practice, adoptive cell therapies are systemically infused via the bloodstream. However, the bone marrow (BM) is the primary location for acute leukemia initiation and maintenance.^6^, ^7^, ^8^ Anatomically, the BM microenvironment confers cellular interactions and signals promoting leukemia initiation, maintenance and progression, as well as drug resistance of leukemic cells.^9^ Current challenges associated to CAR T‐cell treatment in acute leukemia patients might be partially overcome by a rapid and effective CAR T‐cell redirection to the BM. In fact, efficient seeding of systematically infused CAR T cells in the leukemic BM might enhance CAR T‐cell activity and persistence, eventually providing key clinical benefits associated to the potential reduction of the CAR T‐cell dose infused, namely less procedure‐related toxicities, lower production costs, and broader patient's inclusion criteria.

Although several mechanisms regulate the homing of circulating cells to the BM,^10^, ^11^, ^12^ the ability of circulating cells to traffic to the BM initially relies on their robust adherence to the E‐selectin receptor (CD62E) displayed in the vascular endothelium (VE). Adhesive interactions between the E‐selectin receptor and its cognate ligand, tetrasaccharide sialyl‐Lewis X (sLeX), displayed on circulating cells dictate adherence of circulating cells to the VE, the first step of such biological process.^8^ Cell binding activity to E‐selectin receptor is specifically exerted by the sialofucosylated E‐selectin ligands CLA, CD43E, and HCELL resulting from the sLeX instalment (α‐2,3‐sialic acid and α‐1,3‐fucose binding determinants on *N*‐glycans) in the native E‐selectin ligands PSGL1, CD43, and CD44, respectively.^13^, ^14^ Of note, native E‐selectin ligands can be converted into sLeX‐displaying (sialofucosylated) E‐selectin ligands through a straightforward glycan engineering approach involving minimal *ex vivo* cellular manipulation based on a α‐1,3‐fucosyltransferase enzymatic reaction and guanosine diphosphate‐fucose (GDP‐fucose) substrate.^15^, ^16^, ^17^ Such exofucosylation reaction was previously shown to endow BM homing abilities to hematopoietic stem/progenitors cells (HSPCs), mesenchymal stem/stromal cells (MSCs), and immune cells.^15^, ^18^, ^19^

Here, using CD19‐CAR T cells as a working model, we sought (i) to characterize the basal/cell‐autonomous display of sLeX in CAR T‐cells activated with either IL‐2 or IL‐7/IL‐15, and (ii) to assess whether exofucosylation of E‐selectin ligands to enforce sLeX display improves CD19‐CAR T‐cell activity and BM homing. Our results revealed that cell‐autonomous sialofucosylation steadily augments in culture‐ and *in vivo*‐expanded CAR T cells, and that the type of cytokines used during T‐cell activation influences both the cell surface display of sLeX in CD19‐CAR T cells and the CD19‐CAR T‐cell efficacy and persistence *in vivo*. However, enforced sLeX display in E‐selectin ligands by exofucosylation was dispensable for both CD19‐CAR T‐cell activity and BM homing, regardless the cytokines employed for T‐cell expansion. Collectively, glycoengineered sLeX display in CAR T‐cells systemically administered is a dispensable strategy for improved CAR T‐cell function.

## 2 RESULTS

### 2.1 Cell‐autonomous and exofucosylation‐enforced expression of sLeX in CD19‐CAR T cells

**FIGURE 1:**
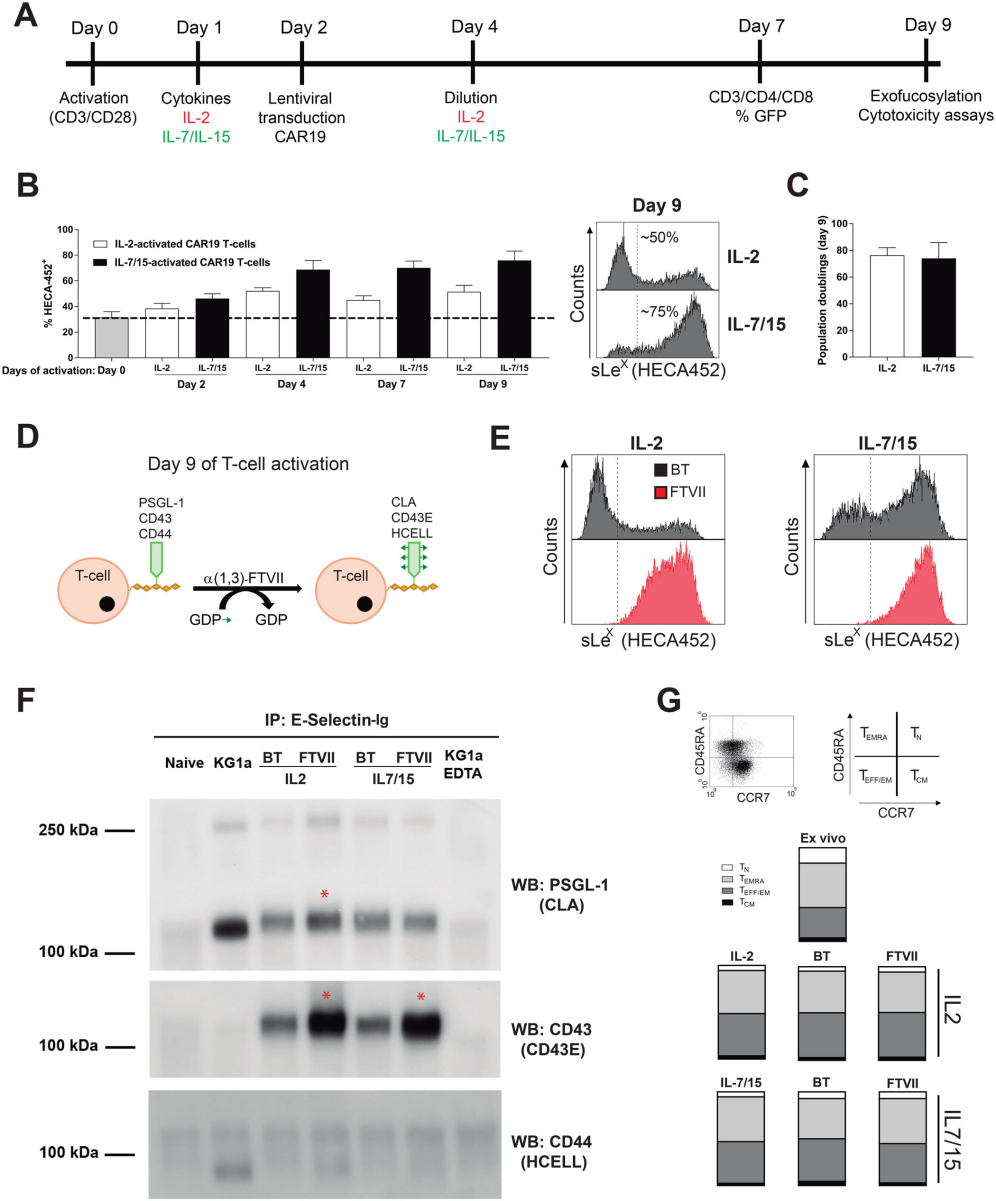
Cell‐autonomous and enforced expression of sLeX in CAR T cells. (A) Experimental design and timing of *ex vivo* CAR T‐cell activation, transduction and exofucosylation. (B) Basal expression and *in vitro* regulation of sLeX (detected by HECA452 MoAb) at the indicated time points in IL‐2‐ or IL‐7/IL‐15‐activated T cells (n = 5 independent donors). Horizontal dotted line depicts the basal expression of HECA452 before T‐ cell activation. Right panels, representative FACS analysis of sLeX (HECA452) at the end of the activation protocol. Vertical dotted lines represent the isotype staining. (C) Very similar T‐cell expansion at the end (day 9) of the *in vitro* culture protocol under both IL‐2‐ and IL‐7/IL‐15‐activation conditions. (D) Cartoon of the *ex vivo* exofucosylation reaction using fucosyltransferase‐VII (FTVII) and GDP‐fucose. Exofucosylation of PSGL‐1, CD43, and CD44 generates the corresponding glycomodified E‐selectin ligands CLA, CD43E, and HCELL, respectively. (E) Representative FACS analysis of sLeX (HECA452) expression in IL‐2‐ or IL‐7/IL‐15‐activated T cells after buffer‐(BT) or FTVII‐treatment (n = 5). (F) Western blot analysis of E‐selectin reactive glycoproteins in naïve T cells, KG1a cells (positive control), and BT or FTVII‐treated CAR T cells. E‐selectin‐Ig immunoprecipitated (IP) glycoproteins from BT or FTVII‐treated CAR T cells were blotted against (PSGL‐ 1, CD43, and CD44). Representative image of n = 3 independent donors. (G) Top panels, a representative FACS plot depicting how TN, TCM, TEFF/EM, TEMRA T‐cell subsets were identified using a CCR7 and CD45RA staining. Bottom panels, relative proportion of TN, TCM, TEFF/EM, TEMRA cell subsets after IL‐2‐ versus IL‐7/IL‐15‐based T‐cell activation or after exofucosylation (FTVII‐ versus buffer treatment)

T cells from peripheral blood (PB) of healthy donors (n = 5) were activated using CD3/CD28 plus either IL‐2 or IL‐7/IL‐15 and transduced on day 2 with CD19‐CAR‐expressing lentivectors (Figure 1A). The levels of sialofucosylation (sLeX display in cell surface) were analyzed by FACS on CAR T cells over the 9‐day activation/expansion period using the HECA452 MoAb, which recognizes sLeX.^20^ Basal expression of sLeX (HECA452+) was found in approximately 31 ± 5% of T cells at day 0 (Figure 1B). Interestingly, *in vitro* T‐cell activation/expansion led to a cell‐autonomous gradual increase in sLeX‐expressing CAR T cells (Figure 1B). Of note, IL‐7/IL‐15 activation consistently rendered higher frequency of sialofucosylated (HECA452+) CAR T cells than IL‐2‐based activation (75 ± 7% vs 50 ± 5% at day 9; Figure 1B), in a T‐cell proliferation/expansion independent manner (Figure 1C). Using a well‐established FTVII‐based exofucosylation reaction^17^ (Figure 1D), 100% of culture‐expanded CAR T cells became HECA452+ within 48 h, regardless the cytokines used during T‐cell activation (Figure 1E). Western Blot (WB) analysis using E‐selectin‐Ig immunoprecipitates clearly identified CD43 (CD43E), and partially PSGL1 (CLA), as the E‐selectin ligands carrying sLeX in exofucosylated CAR T cells (Figure 1F). We next analyzed the phenotype of the expanded T cells using a CCR7 and CD45RA staining, and found that neither the cytokines used nor the exofucosylation reaction affected the T‐cell phenotype (TN, TCM, TEFF/EM, TEMRA) upon 9 days of expansion (Figure 1G). Collectively, although the cytokines used for T‐cell activation influence the level of sLeX display in CAR T cells, cell‐autonomous sialofucosylation gradually increases in culture‐expanded CAR T cells, with up to 80% of CAR T cells being endogenously fucosylated at the end of the *in vitro* expansion.

### 2.2 Exofucosylation enhances neither cytotoxic activity nor homing of CAR T cells *in vitro*

**FIGURE 2:**
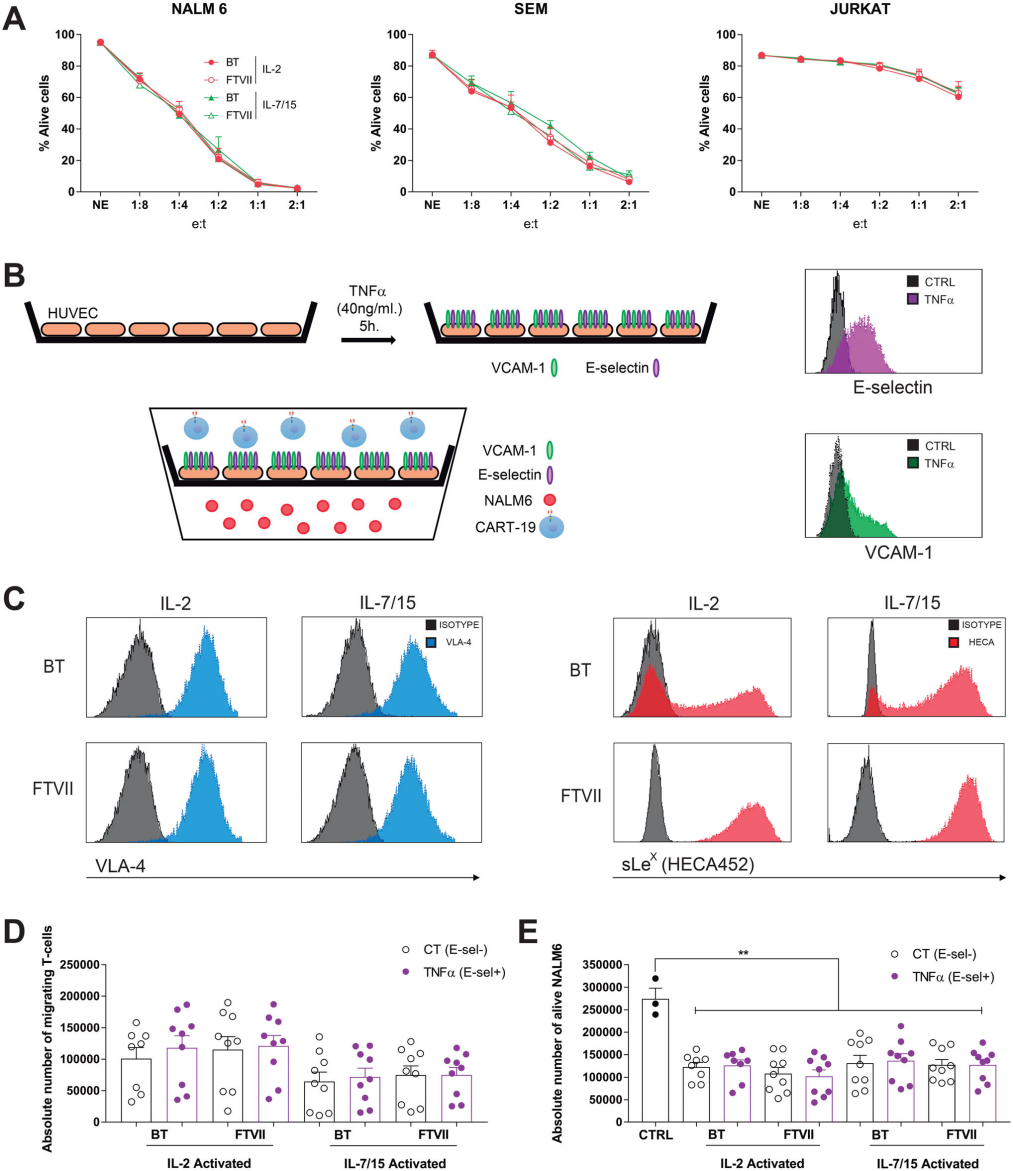
Exofucosylation enhances neither *in vitro* cytotoxicity nor homing of CAR T‐cells on TNF‐α‐activated HUVEC cells. (A) IL‐2‐ and IL‐7/IL‐15‐activated CAR T cells were BT‐ or FTVII‐treated before *in vitro* exposure (24 h) to NALM6, SEM, and Jurkat at the indicated Effector:Target ratio. The alive (7‐AAD‐) eFluor 670+ target cells were determined by FACS (n = 3, independent donors). (B) Cartoon depicting the transwell assay using HUVEC monolayers for analysis of CAR T‐cell (upper) migration toward target cells (bottom). HUVECs were stimulated with TNF‐α‐stimulated for 4 h for expression of VCAM‐1 and E‐selectin expression (right panels). (C) Representative FACS of the expression of the VCAM‐1 ligand VLA‐4 (left panels) and the exofucosylated E‐selectin ligands (sLeX/HECA452‐expressing, right panels) in IL‐2‐ and IL‐7/IL‐15‐activated CAR T cells after BT‐ or FTVII treatment (n = 3). (D,E) Absolute number of migrating BT‐ or FTVII‐treated CAR T cells (D) and alive (7ADD‐) target cells (E) quantified in the bottom chamber after 24 h (n = 6 donors in three independent experiments). CAR T cells were activated either with IL‐2 or IL‐7/IL‐15. The assays were performed using unstimulated or TNF‐α‐stimulated HUVECs. *P < .05, **P < .01, ***P < .001

We first prompted to analyze *in vitro* the cytotoxic activity of exofucosylated CD19‐CAR T cells. The cytotoxicity and specificity of both BT‐ (control) and FTVII‐treated (sialofucosylated) CD19‐CAR T cells were identical in *in vitro* assays against CD19+ (NALM6, SEM) and CD19‐ (Jurkat) cell lines at multiple effector:target ratios (Figure 2A). We next investigated the potential of exofucosylated CD19‐CAR T cells to migrate through TNF‐α‐stimulated Human Umbilical Vein Endothelial Cells (HUVEC) monolayers using standard *in vitro* transwell migration assays (Figure 2B). As expected, the expression of both E‐selectin receptor and VCAM‐1, a key vascular cell adhesion molecule, was upregulated in HUVEC cells upon TNF‐α stimulation, thus, mimicking an activated microvasculature environment^21^, ^22^ (Figure 2B, right panels). Regardless the cytokines used for T‐cell activation, exofucosylation rendered HECA452 expression in 100% of the FTVII‐treated CD19‐CAR T cells while not affecting the expression of the VLA‐4, the putative VCAM‐1 ligand, confirming the specificity of the FTVII treatment (Figure 2C). BT‐ and FTVII‐treated CD19 CAR T cells (upper chamber) showed identical migratory capacity toward target cells (bottom chamber) through either nonstimulated or TNF‐α‐stimulated HUVEC monolayers (Figure 2D), which translated into identical cytotoxicity of target cells by the migrating CAR T cells, in 24 h assays (Figure 2E). Taken together, enforced exofucosylation of CD19‐CAR T cells enhances neither cytotoxicity nor homing *in vitro*.

### 2.3 Exofucosylation enhances neither homing to BM/spleen nor activity/persistence of CAR T cells *in vivo*

We next assessed whether enforcing sLeX display by exofucosylation promotes rapid migration of CD19 CAR T cells to BM and spleen. A sum of 3 × 10^6^ BT‐ or FTVII‐treated CD19‐CAR T cells were i.v. infused in NSG mice previously intra‐BM transplanted with CD19+ target cells, and the ability of CD19‐CAR T cells to colonize the BM and spleen was analyzed as early as 24 and 72 h after (Supporting information Figure S1A). In line with the *in vitro* data, similar numbers of BT‐ and FTVII‐treated CAR T cells were found in PB and BM 24 and 72 h (Figure S1B) after CAR T‐cell infusion, regardless the cytokines used during T‐cell stimulation. These data suggest that, at least as a “stand‐alone” strategy, exofucosylation of CAR T cells does not speed‐up CD19‐CAR T‐cell colonization of BM.

**FIGURE 3:**
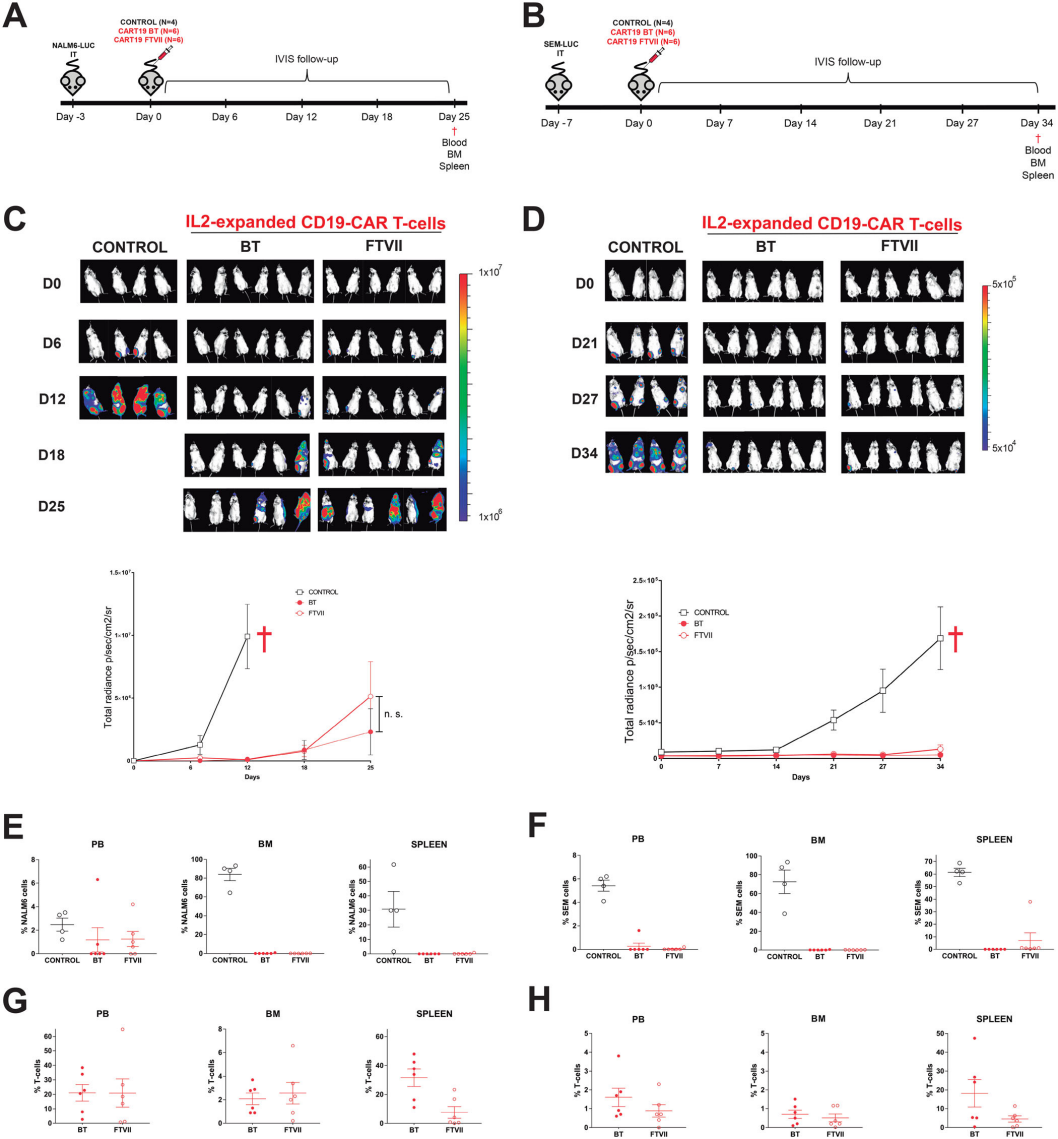
Exofucosylation is dispensable for CAR T‐cell activity and BM homing in both NALM6 and SEM *in vivo* models. (A,B) Schematic of CAR T‐cell activity and homing using NALM6 (A) or SEM (B) as target cells. NSG mice (n = 6 per group) intra‐BM transplanted with 1 × 105 Luc‐expressing NALM6 cells were i.v. infused 72 h later with 3 × 10^6^ BT‐ or FTVII‐treated CAR T cells. NALM6/SEM engraftment was followed weekly by bioluminescence. Target cell and T‐cell engraftment was FACS analyzed in PB, BM, and spleen at sacrifice (for NALM6, day +12 for Mock T cells and day +25 for CAR T‐cells; SEM, day +34). (C,D) IVIS imaging of NALM6 (C) and SEM (D) tumor burden monitored by BLI at the indicated time points. Bottom panels, total radiance quantification (p/s/cm2/sr) at the indicated time points. †: sacrifice. (E,F) NALM6 (E) and SEM (F) engraftment in PB, BM, and spleen at sacrifice in control mice (Mock T‐cells), BT‐ and FTVII‐ treated CAR T‐cells. (G,H) Persisting CAR T cells in PB, BM, and spleen at sacrifice in control mice, BT‐, and FTVII‐treated CAR T cells in the NALM6 (G) and SEM (H) model

**FIGURE 4:**
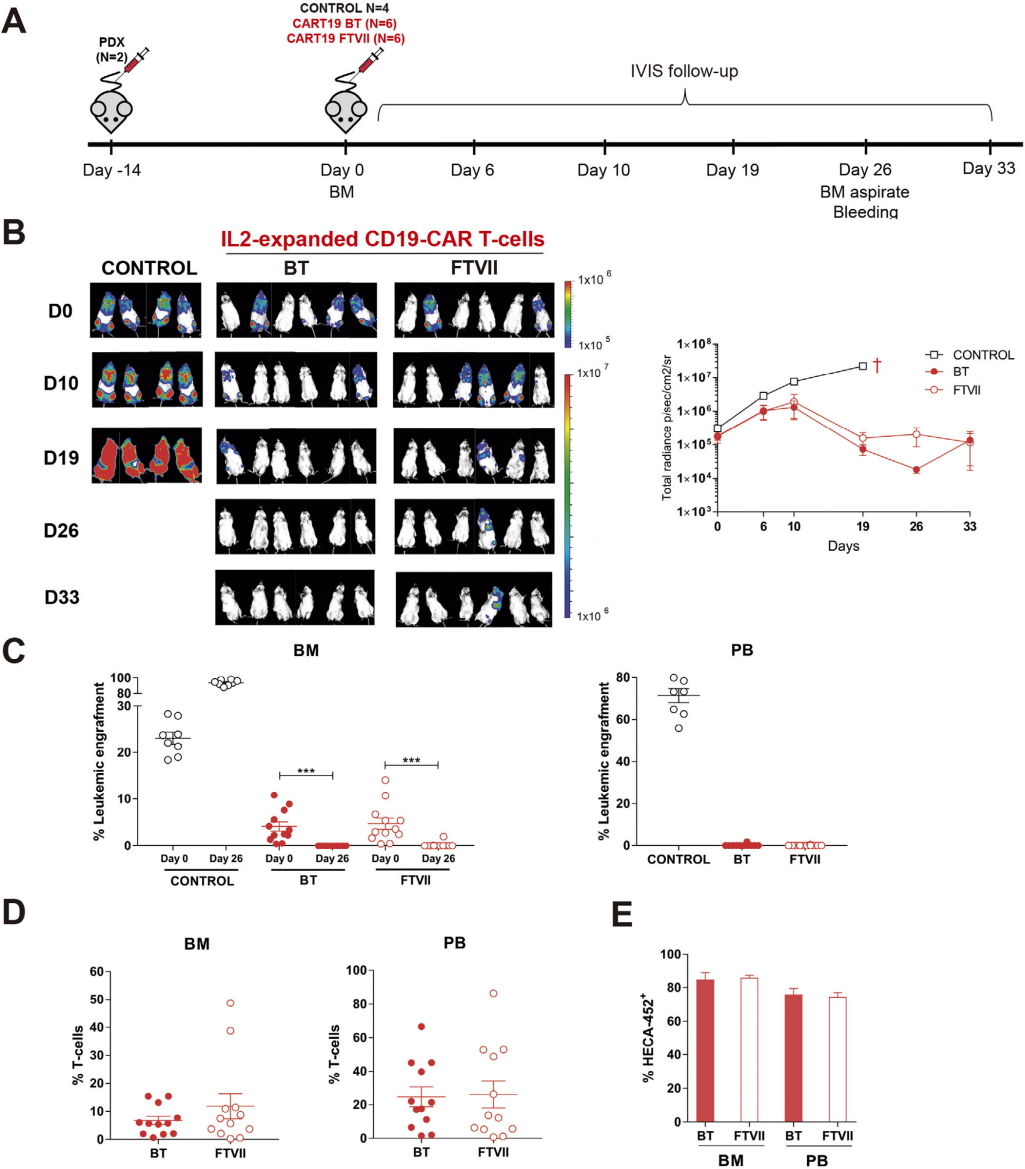
Exofucosylation is dispensable for CAR T‐cell activity and BM homing in B‐ALL PDX models. (A) Schematic of CAR T‐cell activity and homing experiment using two independent B‐ALL PDX (n = 2). NSG mice (n = 6 per group) were i.v. transplanted with 5 × 10^5^ Luc‐expressing PDX cells and 3 × 10^6^ BT‐ or FTVII‐treated 3 × 10^6^ CAR T‐cells were i.v. infused 14 days later, after engraftment was determined by FACS. Mice were distributed equally in each group. PDX engraftment was followed weekly by BLI. Tumor burden and T‐cell engraftment was analyzed in PB and BM at sacrifice (day +33). (B) IVIS imaging of tumor burden monitored by BLI at the indicated time points. Right panel, total radiance quantification (p/s/cm2/sr) at the indicated time points. †: sacrifice. (C) Tumor burden in PB and BM at sacrifice in control (Mock T‐cells) mice, BT‐ and FTVII‐treated CAR T cells. (D) Persisting CAR T cells in PB and BM at sacrifice in control mice, BT‐, and FTVII‐treated CAR T‐cells. (E) Expression of sLeX (HECA452) in BT‐ and FTVII‐treated CAR T cells circulating in PB and present in BM at sacrifice

We next investigated whether exofucosylation endows CD19‐CAR T cells with an enhanced cytotoxic activity or prolonged persistence. Three *in vivo* models using differentially aggressive targets cells were employed (Figures 3 and 4). A sum of 3 × 10^6^ of IL‐2‐expanded BT‐ or FTVII‐treated CD19‐CAR T cells from different donors were i.v. infused in NSG mice 3, 7, or 14 days after transplantation of Luc‐expressing NALM6 (Figure 3A), SEM (Figure 3B), or B‐ALL PDX (n = 2, Figure 4A), respectively. BT‐ or FTVII‐treated CD19‐CAR T cells were equally effective in controlling the leukemia overtime, regardless the target cells used (Figures 3C, D and 4B). FACS analysis of residual target cells at sacrifice revealed identical cytotoxic activity of both BT‐ or FTVII‐treated CD19‐CAR T cells in controlling NALM6 (Figure 3E), SEM (Figure 3F), and PDX B‐ALL (Figure 4C) cells in PB, BM, and spleen. In addition, endpoint analysis of CAR T cells revealed no persistence advantage of FTVII‐treated effector CAR T cells in PB, BM, and spleen (Figures 3G, H and 4D). We then infused limiting doses of BT‐ and FTVII‐treated CAR T cells (in the SEM model) to more accurately assess whether FTVII‐treatment may provide an improved *in vivo* cytotoxic activity of CAR T cells when administered in limited numbers, and found that exofucosylation did not endow CAR T cells with an improved antileukemia effects regardless the cell dose infused (Supporting information Figure S2). We finally measured the frequency of sialofucosylated (HECA452+) CAR T cells in both BM and PB at sacrifice of the B‐ALL PDX models, and found identical levels (∼80%) of sLeX display in both BT‐ and FTVII‐treated CAR T cells (Figure 4E), further indicating cell‐autonomous sialofucosylation of *in vivo*‐expanded CAR T cells.

Of note, the results from these three *in vivo* models were fully reproduced with IL‐7/IL‐15‐activated/expanded CAR T cells (Supporting information Figure S3), further validating that glycoengineered sLeX display in CAR T cells is dispensable for CAR T‐cell activity and persistence, regardless the cytokines used during T‐cell stimulation. However, regardless enforced exofucosylation of CAR T cells, IL‐2‐expanded CD19‐CAR T‐cells displayed a better control of the disease coupled to a higher T‐cell persistence than IL‐7/IL‐15‐expanded CD19 CAR T cells in all the *in‐vivo* leukemia models used (NALM6, SEM, and PDXs, Figures 3 and 4 versus Supporting information Figure S3).

## 3 DISCUSSION

CAR T‐cell therapy has been acclaimed as a revolution in cancer treatment following the impressive results in hematological B‐cell malignancies, especially in refractory/relapse B‐ALL. However, despite the impressive response rates, CD19‐directed adoptive cell immunotherapy is on its infancy, and unfortunately, a large proportion of CD19‐CAR T‐cell‐treated patients eventually progress due to either poor CAR T‐cell persistence and/or disease relapse.^23^, ^24^ Indeed, many studies in the coming years are expected to seed light into key molecular and cellular immunological mechanisms underlying CAR T‐cell biology.^25^ Furthermore, CAR T cells for solid tumors are lagging behind in part because the need to circumvent the physical barriers of the tumor architecture such as subverted tumor vasculature, impediments of CAR T‐cell trafficking, and immunesuppressive microenvironment.^26^ Similarly, the primary location for acute leukemogenesis is the BM, and the BM microenvironment provides leukemic cells with cellular interactions and signals promoting leukemia initiation, progression, and chemoresistance.^6^, ^7^, ^8^, ^9^ However, CAR T cells in patients suffering from acute leukemias are systemically infused via the bloodstream.

Practically all cellular therapies systemically administered to treat hematological malignancies such as transplantation of unmodified or gene therapy‐modified HSPCs,^27^ or infusion of donor unmodified immune cells rely on efficient seeding in the leukemic BM.^28^ Similarly, cell therapy based on MSCs for graft‐versus‐host disease or inflammatory conditions also rely on successful MSC trafficking/homing to the damaged tissue.^29^, ^30^ Here, we have hypothesized that CAR T‐cell immunotherapies (CD19‐CAR as a working model) in acute leukemia patients may also benefit from a rapid and effective redirection of effector cells to BM. Efficient seeding of infused CAR T cells in the leukemic BM might enhance their activity and persistence, eventually providing many clinical benefits associated to the potential reduction in the CAR T‐cell dose to be infused, namely less CRS, lower production costs, and broader patient´s inclusion criteria. Previous studies from several laboratories have suggested that enforced expression *ex vivo* of E‐selectin ligands (exofucosylation) leads to transendothelial migration of systemically administered HSPCs, MSCs, and T cells at E‐selectin‐expressing endothelial beds.^16^, ^19^, ^31^, ^32^, ^33^ Here, we have addressed the role of cell‐autonomous and enforced sialofucosylation (sLeX display) in E‐selectin ligands in the cytotoxic activity and homing ability of systemically administered CD19 CAR T cells. Taking advantage of state‐of‐the‐art *in vitro* assays as well as short‐ and long‐term *in vivo* xenograft models using several B‐ALL cell lines and PDXs, our FACS and biochemical data revealed that cell‐autonomous sialofucosylation steadily increases in culture‐ and *in vivo*‐expanded CAR T cells. In contrast, a study by Mondal et al. has recently shown that *in vitro*‐expanded CAR T cells do not exhibit sLeX expression/E‐selectin binding capacity.^34^ One may attribute such differences to the use of different scFvs, T‐cell activation conditions, biological differences of the donor T‐cells employed (HLA haplotypes, age, comorbilities), etc. We have systematically compared side‐by‐side IL‐2‐ versus IL‐7/IL‐15‐based T‐cell activation conditions, concluding that the cytokines used for T‐cell activation influence the degree of cell‐autonomous sLeX expression in CAR T cells. However, regardless of the cytokines used for T‐cell activation, cell‐autonomous sLeX expression/E‐selectin binding capacity gradually increased in culture‐ and *in vivo*‐expanded CAR T cells. Moreover, identical levels of sLeX expression/sialofucosylation were observed in *in vitro*‐expanded CAR+ and CAR‐T cells, suggesting a CAR‐independent cell‐autonomous sialofucosylation of activated culture‐expanded T cells (data not shown).

Our results using multiple *in vitro* and *in vivo* xenograft models revealed that further enforced sLeX‐installation in E‐selectin ligands improves neither the cytotoxic activity nor BM/spleen homing of vascularly administered CD19‐CAR T cells, regardless of the cytokines used for T‐cell activation. Of note, the T‐cell phenotype was not altered by either the cytokines used for T‐cell expansion or the exofucosylation reaction. This is in line with the reported cell‐autonomous steady sialofucosylation of *in vitro* culture‐expanded CAR T cells prior to *in vivo* infusion. Furthermore, the frequency of HECA452+ CAR T cells was found very similar in xenografts infused with either BT‐ or FTVII‐treated CAR T cells, suggesting that cell‐autonomous sialofucosylation of T cells *in vivo* seems sufficient for proper *in vivo* effector function. A major difference between our experimental design and that by Mondal et al. is that, in our study, CAR T cells were infused in NSG mice previously transplanted with CD19+ target cells, thus, making our *in vivo* model more informative. It should be noted that CAR T cells are not expected to migrate or persist in BM or spleen in the absence of target antigen. Therefore, our xenograft models permit a physiologically more relevant *in vivo* assessment of (i) the trafficking ability of the infused exofucosylated CAR T cells in the presence of target antigen‐expressing leukemic niches, and (ii) the cytotoxic activity of exofucosylated CAR T cells. In a different adoptive immunotherapy context, tumor infiltrated lymphocytes (TILs) have been recently reported to display an increased *in vivo* but also *in vitro* cytotoxic activity upon exofucosylation, suggesting that the enhanced sLeX expression seemed important for the target cell recognition.^35^ Furthermore, the mechanisms for activation/expansion and target cell recognition of TILs clearly differ from those from CAR T cells, further explaining such experimental discrepancies between the distinct effector cells used for adoptive cell immunotherapies. Of note, regardless enforced exofucosylation of CAR T cells, IL‐2‐expanded CD19‐CAR T cells showed a better control of the disease coupled to a higher T‐cell persistence than IL7‐/IL‐15‐expanded CD19‐CAR T cells in all the *in‐vivo* leukemia models employed in the present study (NALM6, SEM, and PDXs), suggesting that adequate T‐cell expansion protocols may benefit the manufacturing and clinical outcome of CAR T cells. Collectively, our results support that the cytokines used during T‐cell activation influence both the degree of cell‐autonomous sialofucosylation and the CD19‐CAR T‐cell efficacy and persistence *in vivo*. However, at least as a “stand‐alone” strategy, glycoengineered exosialofucosylation of E‐selectin ligands seems dispensable for CD19‐CAR T‐cell activity and BM homing in multiple xenograft models regardless of cytokines employed for T‐cell expansion. Which and how alternative cellular and molecular mechanisms regulate the migration of circulating CAR T‐cells to BM needs to be explored in further studies.

## 4 MATERIALS AND METHODS

### 4.1 CD19‐CAR vector, lentiviral production, T‐cell transduction, activation and expansion

Our clinically validated pCCL lentiviral second‐generation CD19CAR backbone containing a human CD8 transmembrane (TM) domain, human 4‐1BB and CD3z endodomains, and a T2A‐GFP cassette has been reported elsewhere.^36^, ^37^ CAR‐expressing viral particles pseudotyped with VSV‐G were generated in 293T cells using standard polyethylenimine transfection protocols, and were concentrated by ultracentrifugation as described elsewhere.^38^ Viral titers were consistently in the range of 10^8^ TU/mL. Peripheral blood mononuclear cells (PBMCs) were isolated from buffy coats from healthy volunteers by using Ficoll–Hypaque gradient centrifugation. Buffy coats were obtained from the Barcelona Blood and Tissue Bank upon institutional review board approval (HCB/2018/0030). T cells were plate‐bound activated with anti‐CD3 and anti‐CD28 antibodies for 2 days and then transduced with CAR‐expressing lentivirus (multiplicity of infection = 10) in the presence of either interleukin‐2 (IL‐2, 50 UI/mL, Mitenyi Biotec) or IL‐7 and IL‐15 (10 ng/mL, Mitenyi Biotec).^38^ Proper CAR expression, T‐cell activation, and expansion was confirmed at the end of the activation period, as previously described.^38^

### 4.2 Exofucosylation reaction

CD19‐CAR T cells expanded for 9 days either with IL‐2‐ or IL‐7/IL‐15 were treated on Hanks' Balanced Salt solution (0.1% human serum albumin and 10 mM HEPES) with GDP‐fucose (Biosynth Carbosynth, Compton, UK) and FTVII (RD Systems). One million cells were incubated in 20 μL of buffer containing 1 mM of GDP‐fucose and 70 μg/mL of purified FTVII enzyme at 37°C for 1 h as previously detailed.^17^ Control cells were incubated in the same solution but without FTVII/GDP‐fucose (buffer‐treated [BT] cells). After the enzymatic reaction, cells were always washed twice with PBS before downstream experiments.

### 4.3 E‐selectin‐Ig immunoprecipitation

CAR T‐cell lysates were prepared using lysis buffer containing 150 mM NaCl, 50 mM Tris‐HCI (pH 7.4), 2% Nonidet P‐40, 2 mM CaCl2, and protease and phosphatase inhibitor cocktails (Roche). When indicated, 5 mM EDTA was added to the lysis buffer as negative control condition. A sum of 5 × 10^6^ cells were pelleted per condition, washed with PBS, and lysed in 500 μL of lysis buffer. Cell lysates were incubated on ice for 15 min and centrifuged at 12 000 *g* for 10 min. Cell lysates were then precleared overnight using protein G‐agarose beads (Roche) and incubated for 2 h at 4°C with 3 μg of murine E‐selectin‐human Fc chimera (“E‐Ig,” R&D Systems), as described.^17^ Agarose beads were then washed twice with lysis buffer, and immunoprecipitated glycoproteins were collected by boiling the beads in the presence of 2‐mercaptoethanol in Laemmli loading buffer. For western blot (WB) analysis, immunoprecipitates were resolved on a 7.5% SDS‐PAGE gel (Bio‐Rad Laboratories) and then transferred onto a polyvinylidene difluoride (PVDF) membrane (Bio‐Rad). The membrane was then blocked with blocking reagent (Chemiluminescence Western Blotting Kit, Roche), and incubated with monoclonal antibodies (MoAb) against PSGL1 (clone KPL1, BD), CD43 (clone IG10, BD), and CD44 (clone 2C5, R&D). Protein bands were detected by chemiluminescence using Lumi‐Light substrate (Chemiluminescence Western Blotting Kit).

#### 4.3.1 *In vitro* cytotoxicity assays

Luciferase (Luc)/GFP‐expressing‐NALM6 cells were kindly provided by Prof. RJ Brentjens (MSKCC, NY). SEM were generated by retroviral transduction and GFP‐based FACS‐selection.^39^ Jurkat were purchased from DSMZ. Target cells were labeled with 3 μM eFluor670 (eBioscience) and incubated with BT‐ and CD19‐CAR T cells at different Effector:Target (E:T) ratios. CAR T‐cell‐mediated cytotoxicity was determined by analyzing the residual alive (7‐AAD‐) eFluor670+ target cells after 24 h CAR T‐cell exposure.

### 4.4 HUVEC transwell assays

HUVEC were maintained in EGM‐2 Endothelial Cell Growth Medium‐2 BulletKit (Lonza, Cultek SLU), as previously described.^40^ Early passage HUVECs were plated on 24‐well Transwell plates (5 μm polycarbonate membrane, 6.5 mm insert), and stimulated with 40 ng/mL of TNF‐α (R&D) for 4 h at 37°C to activate cell surface expression of E‐selectin and VCAM‐1.17 A sum of 2 × 10^5^ of each NALM6 cells and BT‐ or FTVII‐treated CAR T cells were seeded in the bottom and upper chamber, respectively. Twenty‐four hours later, the absolute number of alive (7AAD‐) NALM6 and CAR T cells present in the bottom chamber were quantified using Trucount tubes (BD Biosciences).^38^

### 4.5 *In vivo* NALM6, SEM, and B‐ALL patient‐derived xenograft (PDX) models

Six‐ to twelve‐week‐old nonobese diabetic NOD.Cg‐Prkdc^scid^ Il2rg^tm1Wjl^/SzJ (NSG) mice (Jackson Laboratory) were bred and housed under pathogen‐free conditions in the animal facility of the Barcelona Biomedical Research Park (PRBB). All *in vivo* experimental procedures were performed in compliance with the institutional animal care committee of the PRBB (DAAM7393). Briefly, irradiated (2 Gy) mice (5‐6/condition) were intra‐BM or i.v. transplanted with Luc/GFP‐expressing SEM (1 × 10^5^), NALM6 (1 × 10^5^), or B‐ALL PDX (5 × 10^5^, PDX‐50/PDX‐265) cells and then i.v. infused with 3 × 10^6^ BT‐ or FTVII‐treated CD19‐CAR T cells when engraftment was detectable. An *in vivo* experiment was performed with SEM cells where decreasing doses (2 × 10^6^, 1 × 10^6^, 0.5 × 10^6^, 0.2 × 10^6^) of BT‐ or FTVII‐treated CD19‐CAR T cells were infused. Both IL‐2‐ and IL‐7/IL‐15‐based CAR T‐cell activation protocols were used. Tumor burden was monitored weekly by bioluminescence (BLI) using the Xenogen IVIS 50 Imaging System (Perkin Elmer).^38^ Tumor burden (HLA‐ABC+CD45+CD19+CD22+CD3‐) and CAR T‐cell persistence (HLA‐ABC+CD45+CD3+GFP+) were also quantified by FACS at sacrifice in BM, PB, and spleen.

### 4.6 FACS analysis

Cell staining and FACS analysis were performed as extensively described^38^ using a FACSCanto‐II flow cytometer equipped with FACSDiva software (BDBioscience). Briefly, 0.5 × 10^5^ total cells recovered from *in vitro* or *in vivo* assays were stained in PBS + 2%FBS with the following MoAb: HECA‐452 (CLA)‐BV421, CD62/E‐Selectin‐APC, CD49d/VLA‐4‐PE, VCAM‐1‐APC, HLA‐ABC‐PE, CD45‐BV421, CD3‐PerCP, CD45RA‐AmCyan, CCR7‐PE, CD22‐APC, and CD19‐BV421. IgG1‐APC, IgG1‐PE, and Rat IgM‐BV421 were used as isotype controls. All MoAb were purchased from BD Biosciences. Supporting information Figures S1B and S4 show the gating strategies for T‐cell analysis.

### 4.7 Statistical analysis

Data are shown always from at least three individual donors. At least five animals were used per condition. *P*‐values were calculated by an unpaired two‐tailed Student´s *t‐test* using Prism software (GraphPad).

## AUTHOR CONTRIBUTIONS

Study conception and design: DSM, PM.

Experimentation: DSM, FGA, PR, MV, MC, NT.

Data analysis and interpretation: FGA, DSM, PM.

Technical and primary sample contribution: MP, SRZ, IJ, MJ.

Manuscript writing: DSM, PM.

## CONFLICT OF INTEREST

The authors have nothing to disclose.

## APPROVAL AND CONSENT TO PARTICIPATE

This study was IRB‐approved by the Barcelona Clinic Hospital Ethics Committee (HCB/2019/0450). All *in vivo* procedures were approved by the Animal Care Committee of The Barcelona Biomedical Research Park (HRH‐17‐0029‐P1).

## AVAILABILITY OF DATA AND MATERIAL

The datasets and materials generated in this study are available from the corresponding author on reasonable request.

## Supporting information

Supporting Information

Supporting Information

Supporting Information

Supporting Information

Supporting Information

Supporting Information
