# Supplementary figures and images for: Enforced sialyl‐Lewis‐X (sLeX) display in E‐selectin ligands by exofucosylation is dispensable for CD19‐CAR T‐cell activity and bone marrow homing
Source: Clin Transl Med. 2021 Feb 23;11(2):e280. doi: 10.1002/ctm2.280 (PMC7901721; doi:10.1002/ctm2.280)

A

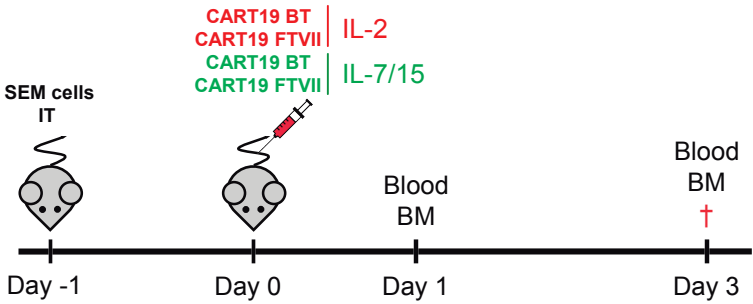

B

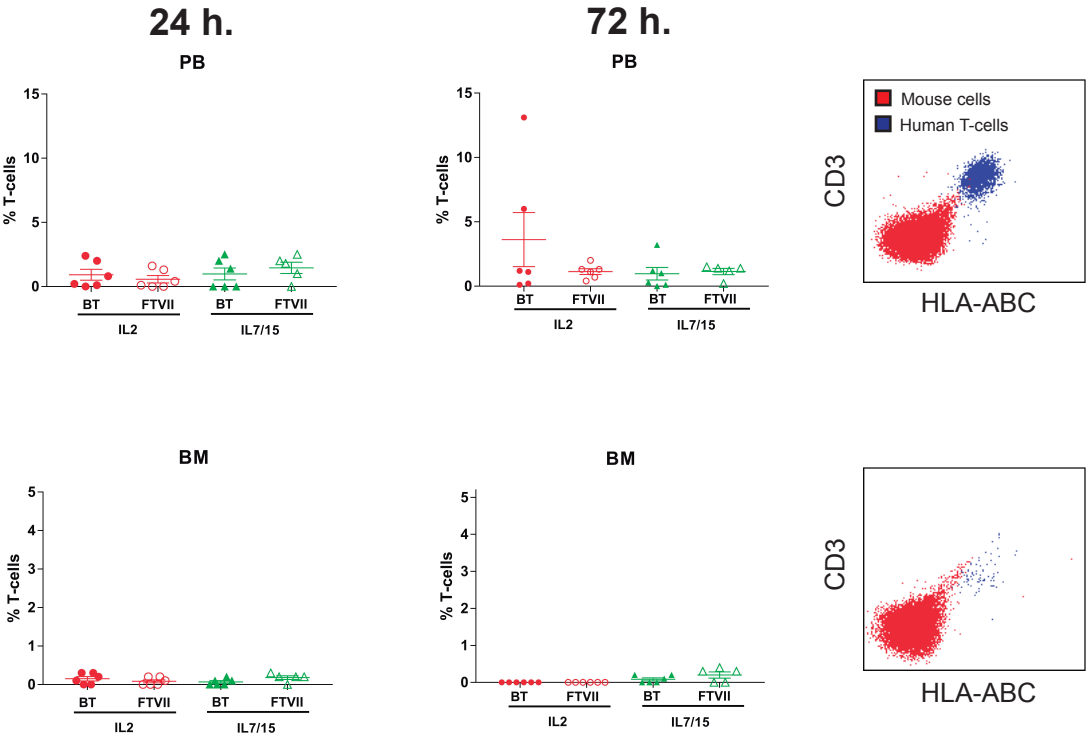

Supplement: Supplementary file 1 — Supporting Information [file CTM2-11-e280-s001.pdf]

A

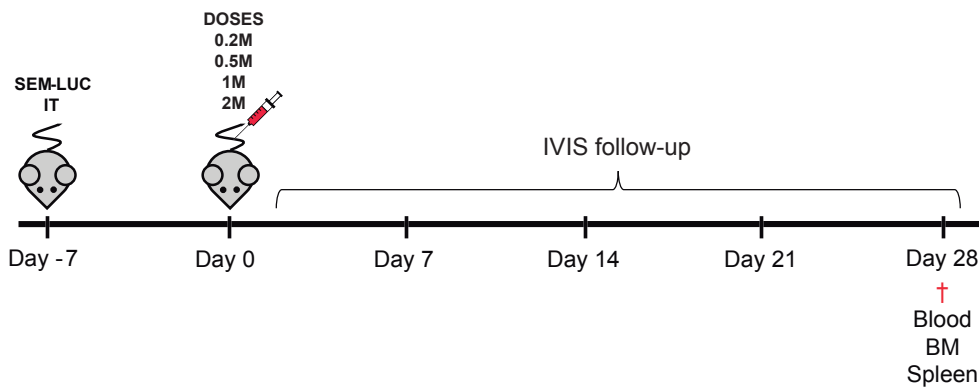

B

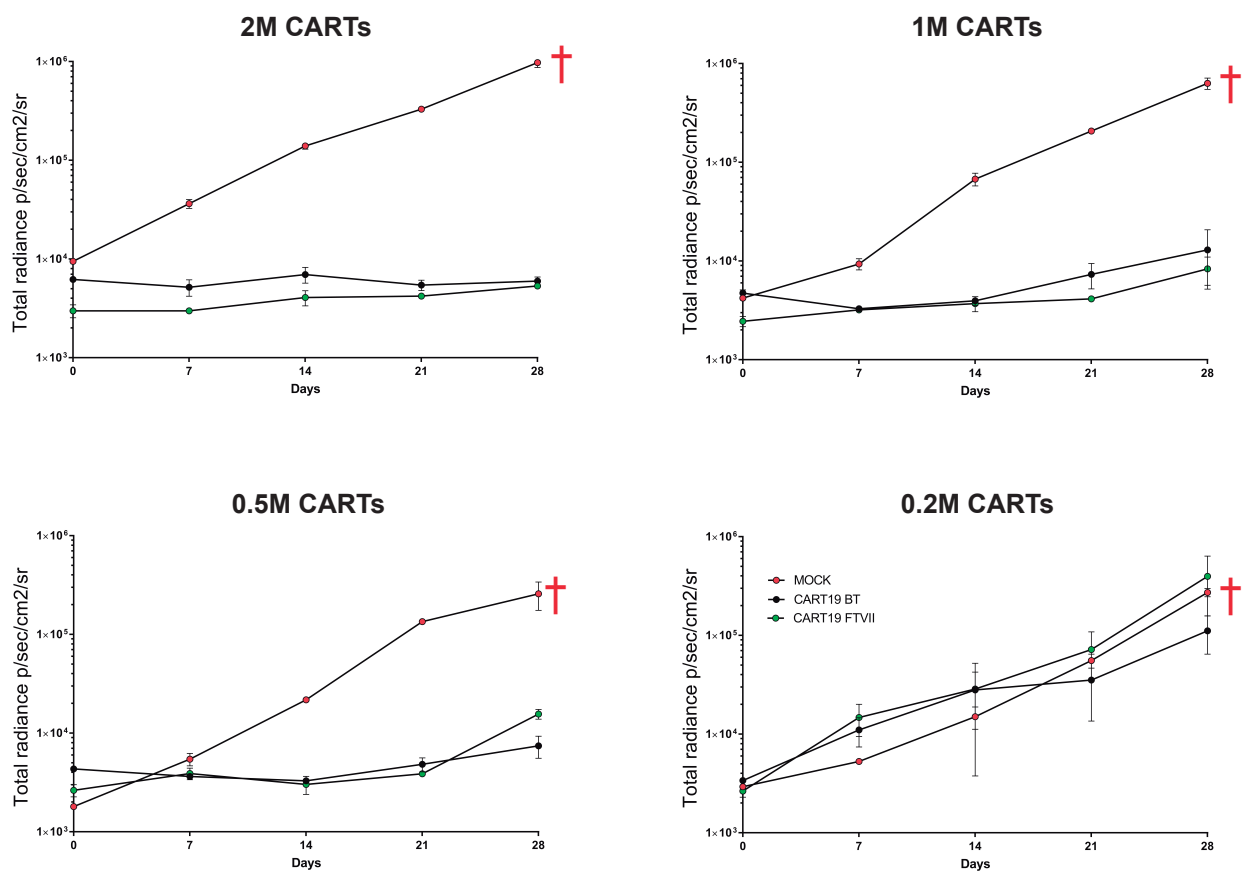

Supplement: Supplementary file 2 — Supporting Information [file CTM2-11-e280-s002.pdf]

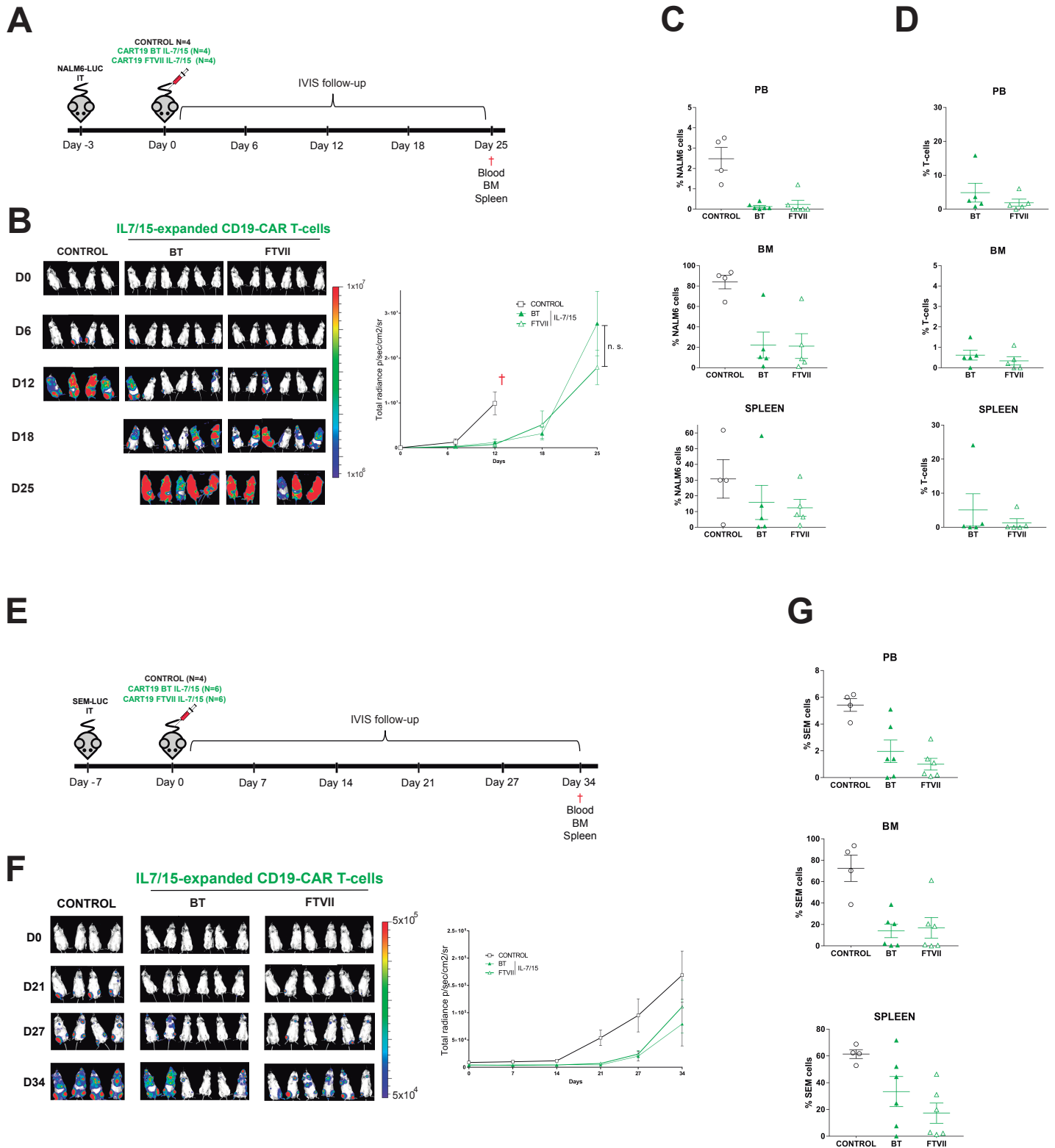

Supplement: Supplementary file 3 — Supporting Information [file CTM2-11-e280-s003.pdf]

H

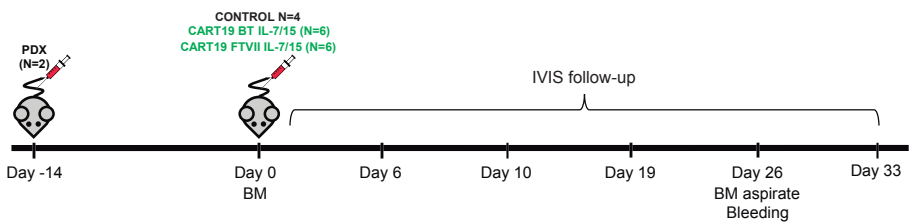

I

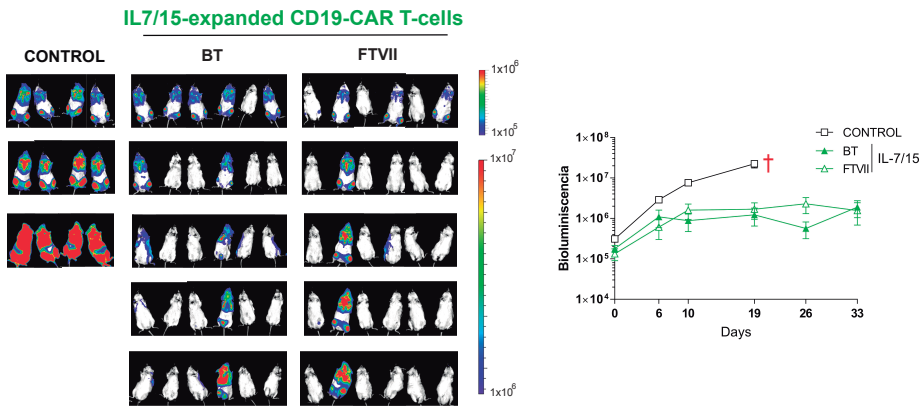

J

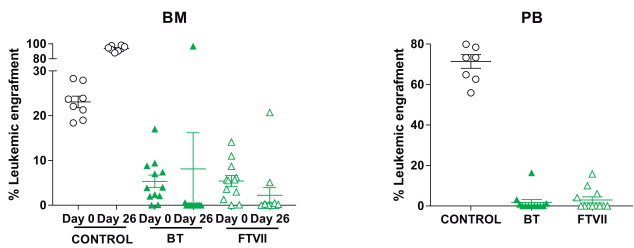

K

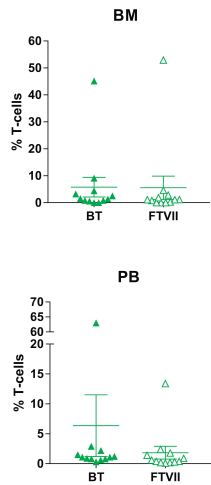

Supplement: Supplementary file 4 — Supporting Information [file CTM2-11-e280-s004.pdf]

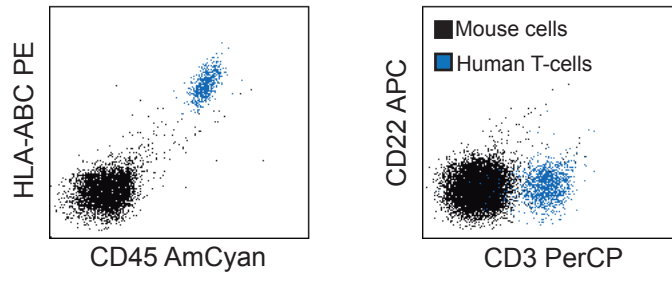

Supplement: Supplementary file 5 — Supporting Information [file CTM2-11-e280-s005.pdf]
