# Supplementary material for: Enforced sialyl‐Lewis‐X (sLeX) display in E‐selectin ligands by exofucosylation is dispensable for CD19‐CAR T‐cell activity and bone marrow homing
Source: Clin Transl Med. 2021 Feb 23;11(2):e280. doi: 10.1002/ctm2.280 (PMC7901721; doi:10.1002/ctm2.280)
Supplement: Supplementary file 6 — Supporting Information [file CTM2-11-e280-s006.pdf]

SUPPLEMENTARY FIGURES

Figure S1. Exofucosylation does not promote rapid CAR T-cell homing to BM. (A) Schematic of CAR T-cell homing short-term (72h) experiment. NSG mice (n=6 per group) were intra-BM transplanted with $1x10^5$ Luc-expressing SEM cells followed, 24h after, by *i.v.* infusion of BT- or FTVII-treated $3x10^6$ CAR T-cell activated with either IL2- or IL7/IL15. CAR T-cells were quantified 72h later in PB, BM and spleen. **(B)** FACS analysis of BT- or FTVII-treated CAR T-cells in PB and BM 24h and 72h after infusion. *Right panels*, representative FACS discriminating between mouse (red) and human cells (blue).

Figure S2. Exofucosylation does not endow CAR T-cells with an improved anti-leukemia effects regardless the cell dose infused. **(A)** Schematic of CAR T-cell activity using SEM as target cells. NSG mice (n=3 per group) intra-BM transplanted with $1x10^5$ Luc-expressing SEM cells were *i.v.* infused 7 days later with decreasing doses ($2x10^6$, $1x10^6$, $0.5x10^6$, $0.2x10^6$) of BT- or FTVII-treated CD19-CAR T-cells. SEM engraftment was followed weekly by bioluminescence up to day +28. **(B)** Total radiance quantification ($p/sec/cm^2/sr$) monitored by BLI at the indicated time points. †: sacrifice.

Figure S3. Identical to Figure 3 for NALM6 and SEM, and Figure 4 for PDXs cells, but using IL7/IL15-activated CAR T-cells.

Figure S4. Gating strategy for T-cell analysis.
